# Alarin alleviated cardiac fibrosis via attenuating oxidative stress in heart failure rats

**DOI:** 10.1007/s00726-021-03005-8

**Published:** 2021-06-05

**Authors:** Jinshuang Li, Hao Ding, Yong Li, Hao Zhou, Wanhong Wang, Yong Mei, Ronglin Zhang

**Affiliations:** 1 Department of Cardiology, Suqian Hospital Affiliated of Xuzhou Medical University, 380 Huanghe South Road, Suqian, 223800 Jiangsu China; 2 Department of Cardiology, The First Affiliated Hospital of Nanjing Medical University, Nanjing, 210029 Jiangsu China; 3 Department of Emergency Medicine, The First Affiliated Hospital of Nanjing Medical University, 300 Guangzhou Road, Nanjing, 210029 Jiangsu China; 4 Department of Cardiology, Nanjing Drum Tower Hospital, The Affiliated Hospital of Nanjing University Medical School, Nanjing, 210008 Jiangsu China

**Keywords:** Alarin, Cardiac fibroblasts, Fibrosis, Oxidative stress, Heart failure

## Abstract

The present study was to explore whether alarin could alleviate heart failure (HF) and attenuate cardia fibrosis via inhibiting oxidative stress. The fibrosis of cardiac fibroblasts (CFs) was induced by angiotensin (Ang) II. HF models were induced by ligation of the left anterior descending artery to cause ischemia myocardial infarction (MI) in Sprague–Dawley rats. Alarin (1.0 nM/kg/d) was administrated by intraperitoneal injection for 28 days. The decreases of left ventricular (LV) ejection fraction (EF), fractional shortening (FS), the maximum of the first differentiation of LV pressure (LV ± d*p*/d*t*_max_) and LV systolic pressure (LVSP), and the increases of LV volume in systole (LVVS), LV volume in diastole (LVVD), LV end-systolic diameter (LVESD) and LV end-diastolic diameter (LVEDD) in MI rats were improved by alarin treatment. The increases in the expression levels of collagen I, collagen III, and transforming growth factor (TGF)-β were inhibited by alarin treatment in CFs and in the hearts of MI rats. The levels of NADPH oxidase (Nox) activity, superoxide anions and malondialdehyde (MDA) levels were increased, and the level of superoxide dismutase (SOD) activity was reduced in Ang II-treated CFs, which were reversed by alarin. Nox1 overexpression reversed the effects of alarin on attenuating the increases of collagen I, collagen III and TGF-β expression levels induced by Ang II in CFs. These results indicated that alarin improved HF and cardiac fibrosis via inhibiting oxidative stress in HF rats. Nox1 played important roles in the regulation of alarin effects on attenuating CFs fibrosis induced by Ang II.

## Introduction

Heart failure, a complex syndrome due to structural or functional cardiac disorders, is characterized by forming interstitial fibrosis of heart, including increases in the expression of collagen I, collagen III and transforming growth factor-β (TGF-β) (Liu et al. 2018; Wang et al. 2019a; Yue et al. 2017). Coronary artery ligation-induced HF rats showed markedly impaired cardiac function and hemodynamics (Gan et al. 2014). Fibrosis in the heart is an important driver of disease progression of chronic heart failure (CHF) (Tarone et al. 2014), and excessive cardiac fibrosis causes large infarct scars, leading to cardiac dysfunction and cardiac dilatation (Kong et al. 2014; Heineke and Molkentin 2006). Cardiac fibroblasts (CFs) are currently considered as the main source of cardiac fibrosis in response to ischemic injury (Tallquist and Molkentin 2017). CFs played important roles in postinfarction remodeling, which can ultimately result in pathological cardiac fibrosis and HF (Philip et al. 2019). Cardiac fibrosis is a hallmark of HF, and there is no effective drug to treat it.

Alarin, a bioactive peptide consisted of 25 amino acids, was identified as the fourth member of the galanin peptide family, just next to galanin, galanin message-associated protein (GMAP) and galanin-like peptide (GALP) (Fang et al. 2020; Miko et al. 2014). Alarin was discovered as an alternate transcript of the GALP gene (Santic et al. 2006, 2007), and can be detected in circulating blood (Hu et al. 2019). It showed that galaninergic system can be considered a promising target to reduce energy dysregulation and attenuate oxidative damage in myocardial injury induced by ischaemia/reperfusion (I/R) of rats (Studneva et al. 2019b). An agonist of galanin receptors GalR1-3 can attenuate doxorubicin-induced cardiotoxicity (Studneva et al. 2019a). Alarin has no detectable affinity toward any of the known three galanin receptor subtypes (Santic et al. 2007; Boughton et al. 2010), therefore its similar effects cannot be expected. Whether alarin could alleviate heart failure and cardiac fibrosis is not well known.

Oxidative stress is often detected and considered to play key roles in pathological cardiac remodeling and the transition to cardiac failure (Sag et al. 2014; Madamanchi and Runge 2013). Angiotensin (Ang) II-indued collagen production is mediated via reactive oxygen species (ROS) generation in adult rat cardiac fibroblasts (CFs). Ang II activates ROS-sensitive kinases that are critical in regulating fibrotic remodeling of the heart (Ohtsu et al. 2005). N-terminal galanin fragment 2–15 (G) decreased H9C2 cells apoptosis and mitochondrial ROS production stimulated by hypoxia and reoxygenation conditions (Pisarenko et al. 2017). However, whether alarin administration attenuated cardiac fibrosis in MI-induced HF rats via inhibiting oxidative stress is not well known. The current study was designed to explore the anti-fibrosis effects of alarin in MI-induced HF rats, and whether oxidative stress was involved in the effects of alarin on alleviating HF.

## Materials and methods

### Animals

Experiments were carried out using 150–180 g male Sprague–Dawley (SD) rats (Vital River Biological Co., Ltd, Beijing, China). The rats were kept in a temperature-controlled room on a 12 h light–dark cycle with free access to standard chow and tap water. All procedures were approved by the Experimental Animal Care and Use Committee of Nanjing Medical University and were conducted in accordance with the Guide for the Care and Use of Laboratory Animals (NIH publication No. 85-23, revised 1996).

### Myocardial infarction model

The myocardial infarction (MI) rats were induced by coronary artery ligation with sterile techniques as previously reported (Gan et al. 2011). Briefly, the rats were anesthetized with sodium pentobarbital (50 mg/kg, i.p.), and randomly subjected to the ligation of the left anterior descending coronary artery and sham-operated (Sham) groups. The heart was exposed through a left intercostal thoracotomy, and left coronary artery was looped by a single nylon suture (7-0). Finally, the heart was quickly repositioned into the chest. The Sham rats were treated the same as the coronary ligation rats except that their coronary arteries were not ligated. One day later, the rats were randomly assigned to four groups, including sham + saline group, MI + saline group, sham + Alarin group, and MI + Alarin group. Alarin rat (1.0 nM/kg/d in 300 μl saline, 026–33, Phoenix Pharmaceuticals, CA, USA) was administrated by intraperitoneal injection for 28 days, rats in the sham group and the MI group received the same volume of saline. In another experiment, an alarin antagonist alarin 6-25 Cys (Wang et al. 2020; Fraley et al. 2013) (Ala6-25 Cys, 2.0 nM/kg/d in 300 μl saline, GL Biochem, Shanghai, China), or galanin receptor antagonists M35 (2.0 nM/kg/d in 300 μl saline, American Peptide Co., CA, USA) or C7 (2.0 nM/kg/d in 300 μl saline, American Peptide Co.) were administrated by intraperitoneal injection following alarin.

### Echocardiography

Twenty-eight days after coronary ligation, transthoracic echocardiography was performed using an ultrasound system (VisualSonics, Toronto, Canada) with a 21-MHz probe under isoflurane anesthesia (2.0–3.0%). The left ventricular end-systolic diameter (LVESD), LV end-diastolic diameter (LVEDD), LV volumes in diastole (LVVd) and LV volumes in systole (LVVs) were measured. The LV ejection fraction (EF), and fractional shortening (FS) were calculated. Measurements were averaged over three consecutive cardiac cycles. Two days later, hemodynamic monitoring was determined.

### Hemodynamic monitoring

Rats were anesthetized with isoflurane (2.0–3.0%). Conductance micromanometer catheter (1.4F, Millar Instruments, TX, USA) was inserted into the LV chamber via the right carotid artery across the aortic valve. The left ventricle systolic pressure (LVSP), and LV end-diastolic pressure (LVEDP), maximum of the first differentiation of LV pressure (LV + d*p*/d*t*) and decline (LV − d*p*/d*t*), were obtained with a PowerLab data acquisition system (AD Instruments, Sydney, Australia).

### Cardiac sample collection

After hemodynamic monitoring determination, the rats were euthanasia by cervical dislocation after anesthetized with 2.5% isoflurane. The heart was removed after phosphate-buffered saline (PBS, Biochannel Biotechnology Co., Ltd, Nanjing, China) perfusion. LV was collected to use in the next experiments. Approximately 50 mg LV sample was used to isolate the RNA or protein.

### Sirius red staining

Cardiac sections (5 µm) were examined by Sirius red staining (Service Biological Technology Co., Ltd, Wuhan, China) according to the manufacture instruments to measure the fibrosis of cardiomyocytes. Three to five random fields (around 30–50 cells per field) were selected from each of three sections from each animal for observation under a light microscope (Carl Zeiss GmbH, Oberkochen, Germany). Images were analyzed using Image-Pro Plus software (Media Cybernetics, Inc., MD, USA).

### Culture of cardiac fibroblasts

Rat cardiac fibroblasts (CFs) were isolated from 1 to 3 days old SD rats. Briefly, CFs were separated from cardiomyocytes by gravity separation and grown to confluence on 10 cm cell culture dishes with growth media (DMEM including 10% FBS, 1% penicillin and 1% streptomycin) at 37 °C in humid air with 5% CO_2_ and 95% O_2_. CFs from the second passage were used for the subsequent experiments. CFs were incubated with angiotensin II human (Ang II, A9525, 10^–6^ M, Sigma, MO, USA) for 24 h to induce the fibrotic phenotype. CFs were assigned to four groups, including PBS group, Ang II group, alarin (10 nM) group, Ang II + alarin (10 nM) group. Alarin and Ang II were devolved in PBS and added into the growth media in 6 cm plate (10^5^ cells/plate) at the same time.

### Quantitative real time-PCR (qRT-PCR)

The total RNA in samples was extracted with Trizol (Ambion, TX, USA). cDNA was extracted from the RNA with reverse transcription using random primers in a total volume of 10 μL according to the instructions of the PrimeScript™ RT Master Mix (Takara, China). All cDNA were stored at − 70 °C before used. Collagen I, collagen III and transforming growth factor (TGF)-β mRNA levels were determined with SYBR Green I fluorescence (Invitrogen; Thermo Fisher Scientific, Inc). All samples were amplified in triplicates for 45 cycles in a 384-well plate. GAPDH was used as the internal controls for mRNA (Yang et al. 2020). Relative expression levels were quantified using the 2^−∆∆*C*q^ method. The primers are shown in Table 1.

**Table 1:** List of utilized primers for qRT-PCR

Gene	Species	Forward primer	Reverse primer
Collagen I	Rat	TCAAGATGGTGGCCGTTAC	CTGCGGATGTTCTCAATCTG
Collagen III	Rat	CGAGATTAAAGCAAGAGGAA	GAGGCTTCTTTACATACCAC
TGF-β	Rat	CAGGGAGTAAGGGACACGA	ACAGCAGTTAGGAACCCAGAT
GAPDH	Rat	GGCACAGTCAAGGCTGAGAATG	ATGGTGGTGAAGACGCCAGTA

*TGF-β* transforming growth factor-β, *GAPDH* glyceraldehyde-3-phosphate dehydrogenase

### Western blotting

The samples were sonicated in RIPA lysis buffer (BioChannel Biotechnology Co., Ltd.) and homogenized. The debris was removed by centrifugation at 12,000×*g* for 10 min at 4 °C and the supernatant was collected. Subsequently, about 30–40 μg protein was separated by gel electrophoresis, transferred to PVDF membrane The membrane was blocked with 5% skimmed milk powder at room temperature for 1 h and probed with primary antibodies overnight at 4 °C against collagen I, collagen III, TGF-β and Nox1 (Abcam, MA, USA). Then, a secondary antibody (Abcam) was added and incubated at room temperature for 1 h and GAPDH (1 Abcam) was used as an internal control. The bands were visualized via ECL (Beyotime, Shanghai, China). Images were analyzed using Image-Pro Plus software (XRayScan; CAD/CAM Services, Inc.).

### NADPH oxidases 1 overexpression

Recombinant adenoviral vectors harboring green fluorescent protein (Ad-GFP) or NADPH oxidases 1 (Ad-Nox1) were obtained from Genechem (Shanghai, China). Adenovirus (10^8^ TU/ml) was added to the growth media.

### Measurement of Nox activity

The Nox activity in the CFs was measured by enhanced lucigenin chemiluminescence. Briefly, the CFs were sonicated and homogenized. The debris was removed by centrifugation at 12,000×*g* for 10 min at 4 °C and the supernatant was collected. NADPH (100 μM) was added to the supernatant as a substrate to react with Nox and generate superoxide anions. The light emission produced by the reaction of lucigenin (5 μM) with superoxide anions was measured with a microplate reader (BioTek, VT, USA) once every minute for 10 min. The values represented the Nox activity and were expressed as the mean light units (MLU) per minute per milligram of protein.

### Measurement of superoxide anions

Superoxide anions level in the CFs was determined by lucigenin-derived chemiluminescence. Briefly, the CFs were sonicated and homogenized. The debris was removed by centrifugation at 12,000×*g* for 10 min at 4 °C and the supernatant was collected. The reaction with superoxide anions was started by adding dark-adapted lucigenin (5 μM) to each sample to cause photon emission, which was measured with a microplate reader (BioTek, VT, USA) once every minute for 10 min. The values representing the superoxide anions level were expressed as the MLU per minute per milligram of protein.

### Malondialdehyde level in the heart

The CFs samples were homogenized in lysis buffer (Thermo Fisher Scientific, MA, USA). The malondialdehyde (MDA) level was determined using an ELISA kit (USCN Business Co., Ltd., Wuhan, China) following the manufacturer’s instructions.

### Superoxide dismutase (SOD) activity level

The CFs samples were collected and homogenated. Superoxide dismutase (SOD) measurement was performed according to the manufacturer’s instructions (Jiancheng Bioengineering Institute, Nanjing, China) using a microplate reader (BioTek, VT, USA).

### Statistical analyses

Data were presented as the mean ± standard error of the mean (SEM) and were analyzed using GraphPad Prism 7.0 (GraphPad software Inc., CA, USA). Statistics were completed using one-way ANOVA, followed by Bonferroni test for post hoc analysis when multiple comparisons were made. A two-tailed *p* value < 0.05 was considered statistically significant.

## Results

### Effects of alarin on cardiac function and fibrosis in HF rats

EF (29.72 ± 2.11 vs. 64.81 ± 3.17) and FS (19.72 ± 1.61 vs. 31.80 ± 1.64) were reduced in MI-induced HF rats, EF and FS were increased by 50.14% and 69.42% after alarin treatment in MI rats. MI induced the increases in LVVS (81.80 ± 8.02 vs. 23.63 ± 2.99 μl), LVVD (106.50 ± 7.60 vs. 52.20 ± 4.41 μl), LVESD (9.43 ± 1.09 vs. 2.01 ± 0.23 mm) and LVEDD (12.71 ± 1.35 vs. 4.43 ± 0.45 mm), which were reversed after alarin. LV + d*p*/d*t*_max_ (2351.67 ± 123.97 vs. 3365.53 ± 135.18 mm Hg/s) and LVSP (106.43 ± 2.91 vs. 132.11 ± 2.19 mm Hg) were reduced in MI rats, and these decreases were enhanced 68.96% and 66.15% by alarin treatment, respectively. LVEDP was increased in the HF rats (8.33 ± 0.70 vs. 3.36 ± 0.44 mm Hg), which was reversed 64.99% by alarin (Table 2).

**Table 2:** Echocardiographic examination of the left ventricular function

Variables	Sham + Saline	MI + Saline	Sham + Alarin	MI + Alarin
EF (%)	64.81 ± 2.11	29.72 ± 3.17*	65.80 ± 2.17	47.36 ± 3.52^#^
FS (%)	31.80 ± 1.64	19.72 ± 1.61*	33.84 ± 1.68	28.19 ± 1.57^#^
LVESD (mm)	2.01 ± 0.23	9.43 ± 1.09*	2.16 ± 0.30	4.15 ± 0.58^#^
LVEDD (mm)	4.43 ± 0.45	12.71 ± 1.35*	4.85 ± 0.47	6.50 ± 0.86^#^
LVVS (μl)	23.63 ± 2.99	81.80 ± 8.02*	30.00 ± 4.45	44.40 ± 6.31^#^
LVVD (μl)	52.20 ± 4.41	106.50 ± 7.60*	49.00 ± 3.51	68.50 ± 6.60^#^
LV + d*p*/d*t*_max_ (mm Hg/s)	3365.53 ± 135.18	2351.67 ± 123.97*	3474.96 ± 125.03	3050.83 ± 168.48^#^
LVSP	132.11 ± 2.19	106.43 ± 2.91*	131.35 ± 2.25	123.47 ± 3.16^#^
LVEDP	3.36 ± 0.44	8.33 ± 0.70*	3.62 ± 0.44	5.10 ± 0.49^#^

The results are expressed as the mean ± SEM of 10 rats per group

*EF* ejection fraction, *FS* fractional shortening, *LV* left ventricular, *LVESD* LV end-systolic diameter, *LVEDD* LV end-diastolic diameter, *LVVD* LV volume in diastole, *LVVS* LV volume in systole

^*^p < 0.05 versus the Sham + Saline group

^#^*p* < 0.05 versus the MI + Saline group

### Effects of alarin on cardiac fibrosis in HF rats

**Fig. 1:**
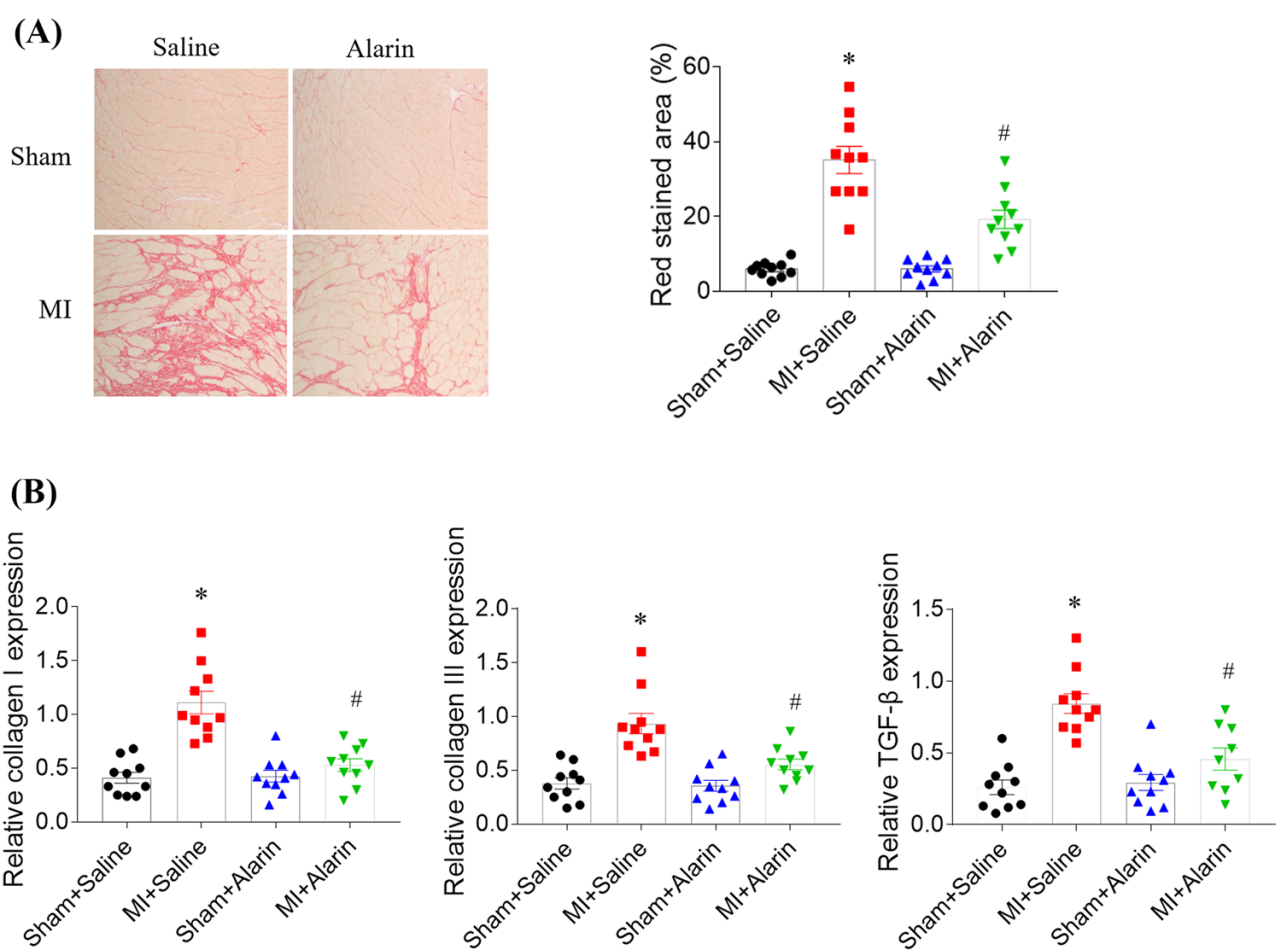
Alarin improved cardiac fibrosis in myocardial infarction (MI) induced heart failure (HF) rats. **A** alarin alleviated cardiac fibrosis in MI-induced HF rats. **B** alarin inhibited the increases of collagen I, collagen III, and transforming growth factor (TGF)-β in the heart of MI rats. The results are expressed as mean ± SEM. *N* = 10 for each group. ^*^p < 0.05 versus the Sham + Saline group; ^#^*p* < 0.05 versus the MI + Saline group

The fibrosis of the heart in MI rat was increased, which was attenuated 54.44% by alarin treatment (Fig. 1A). The expression levels of collagen I, collagen III and TGF-β were increased in the heart of MI-induced HF rats, and these increases were alleviated by alarin (Fig. 1B).

### Effects of alarin and galanin receptor antagonists

**Fig. 2:**
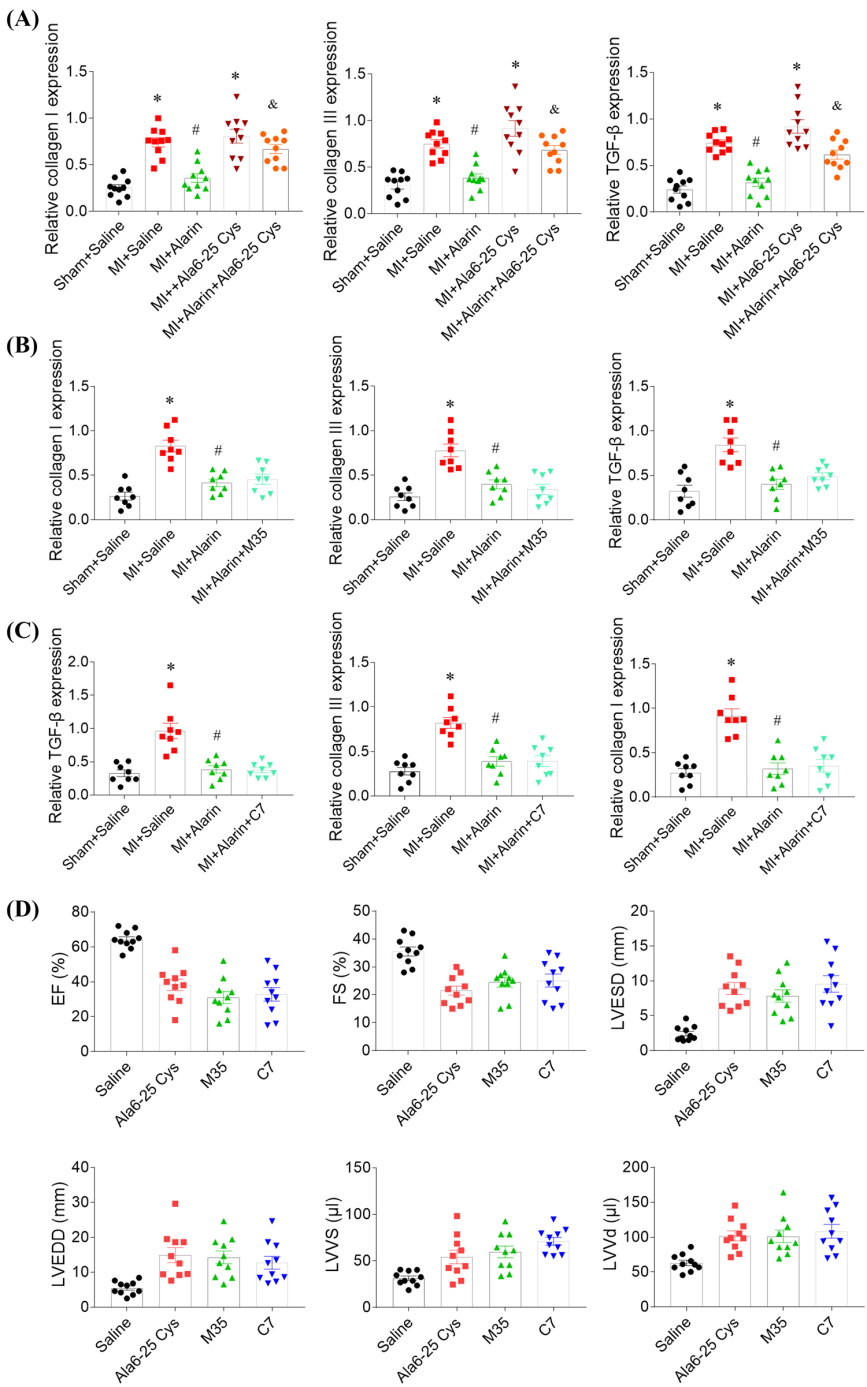
Effects of alarin and galanin receptor antagonists. **A** Alarin 6-25 Cys (Ala6-25 Cys), an antagonist of alarin, reversed the attenuating effects of alarin on the increases of collagen I, collagen III and transforming growth factor (TGF)-β in the heart of myocardial infarction (MI) rats. **B** Galanin receptor antagonist M35 had no effect on the roles of alarin in inhibiting increases of collagen I, collagen III, and TGF-β in the heart of MI rats. **C** Galanin receptor antagonist C7 had no effect on the roles of alarin in inhibiting increases of collagen I, collagen III, and TGF-β in the heart of MI rats. **D** Alarin and galanin receptor antagonists alleviated cardiac dysfunction in myocardial infarction (MI) induced HF rats. The results are expressed as mean ± SEM. *N* = 8 for each group. ^*^p < 0.05 versus the Sham + Saline (**A**, **B** and **C**) or Saline (**D**) group; ^#^*p* < 0.05 versus the MI + Saline group; ^&^*p* < 0.05 versus the MI + Alarin group

Ala6-25 Cys, an antagonist of alarin, reversed the attenuating effects of alarin on the levels of collagen I, collagen III and TGF-β in the heart of MI-induced HF rats. Ala6-25 Cys didn’t further increase the expressions of collagen I, collagen III and TGF-β in the heart of MI rats (Fig. 2A). Galanin receptor antagonist M35 had no effect on the roles of alarin in inhibiting the increases of collagen I, collagen III, and TGF-β in the heart of MI rats (Fig. 2B). Galanin receptor antagonist C7 had no effect on the roles of alarin in inhibiting the increases of collagen I, collagen III, and TGF-β in the heart of MI rats (Fig. 2C). Ala6-25 Cys, M35 and C7 reduced EF and FS of rats compared with the saline group. LVESD, LVEDD, LVVS and LVVD were increased in the rats of treating with Ala6-25 Cys, M35 and C7 (Fig. 2D).

### Effects of alarin on CFs fibrosis induced by Ang II

**Fig. 3:**
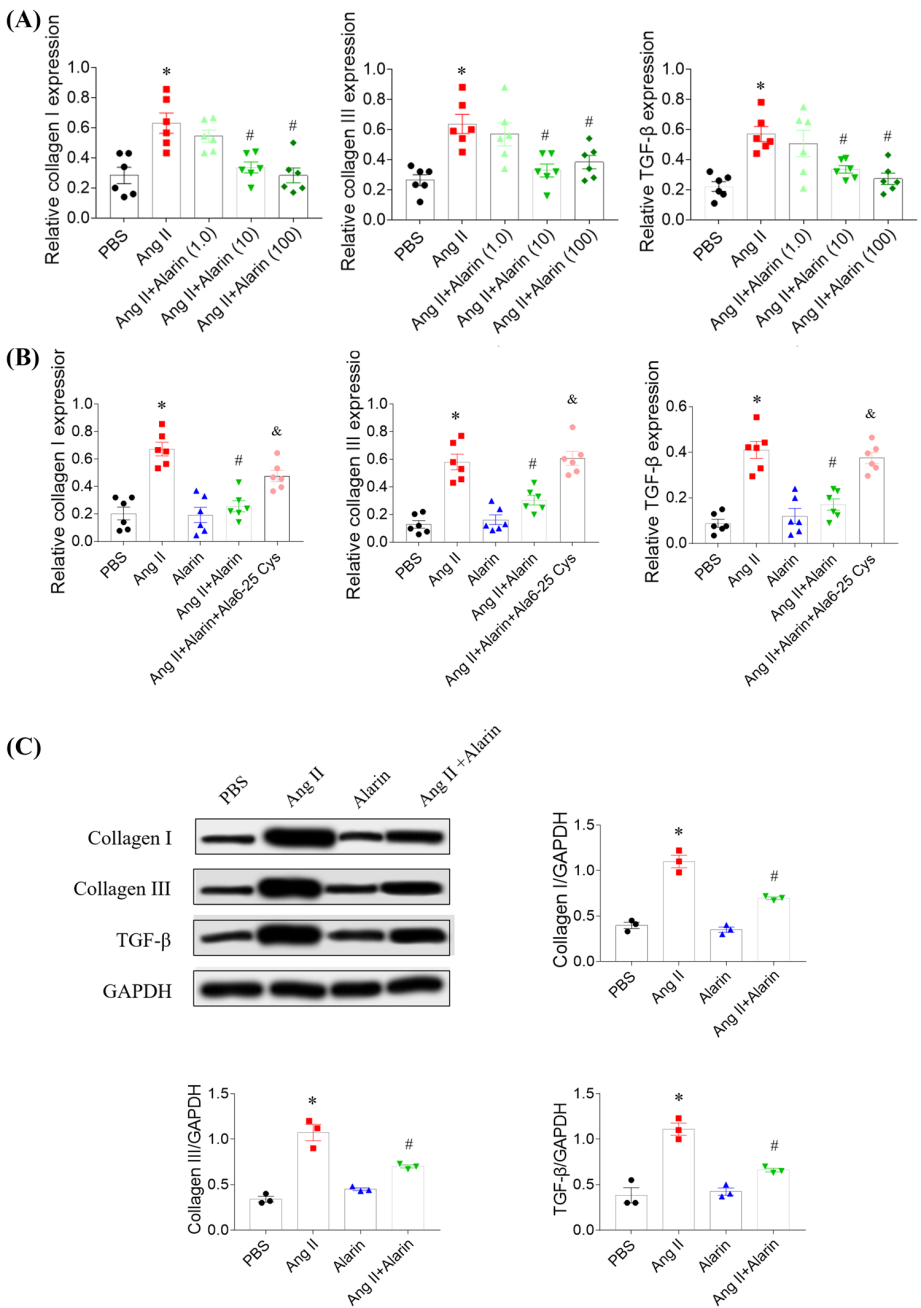
Alarin alleviated cardiac fibroblasts (CFs) fibrosis induced by angiotensin (Ang) II. **A** Low dose had no effect, and middle and high doses of alarin inhibited the increases of collagen I, collagen III and transforming growth factor-β (TGF-β) mRNA levels in CFs induced by Ang II. **B** Alarin 6-25 Cys (Ala6-25 Cys), an antagonist of alarin, reversed the attenuating effects of alarin on the increases of collagen I, collagen III and transforming growth factor (TGF)-β induced by Ang II in CFs. **C** alarin attenuated the increases of collagen I, collagen III and TGF-β protein levels in CFs induced by Ang II. The results are expressed as mean ± SEM. ^*^p < 0.05 versus the PBS group; ^#^*p* < 0.05 versus the Ang II group

Treatment with low dose alarin (1.0 nM) had no effect on the increases of collagen I, collagen III and TGF-β induced by Ang II in CFs. Both the middle (10 nM) and high (100 nM) doses of alarin inhibited the increases of collagen I, collagen III and TGF-β induced by Ang II in CFs (Fig. 3A). Middle dose of alarin (10 nM) was selected in the following experiments.

Ala6-25 Cys reversed the attenuating effects of alarin on the increases of collagen I, collagen III and TGF-β induced by Ang II in CFs (Fig. 3B). The protein expression levels of collagen I, collagen III and TGF-β in CFs were heightened treated with Ang II, which was revised by alarin (Fig. 3C).

### Effects of alarin on oxidative stress

Nox activity level was increased in the heart of MI rats (18.11 ± 1.61 vs. 8.21 ± 0.82 MLU/min/mg protein). Alarin alleviated the increase of Nox activity level in the heart of MI rats (10.89 ± 0.95 vs. 18.11 ± 1.61 MLU/min/mg protein). The increase in the level of superoxide anion in the heart of MI rats (17.56 ± 2.18 vs. 7.66 ± 1.01 MLU/min/mg protein) was attenuated after alarin administration (11.85 ± 0.97 vs. 17.56 ± 2.18 MLU/min/mg protein).

**Fig. 4:**
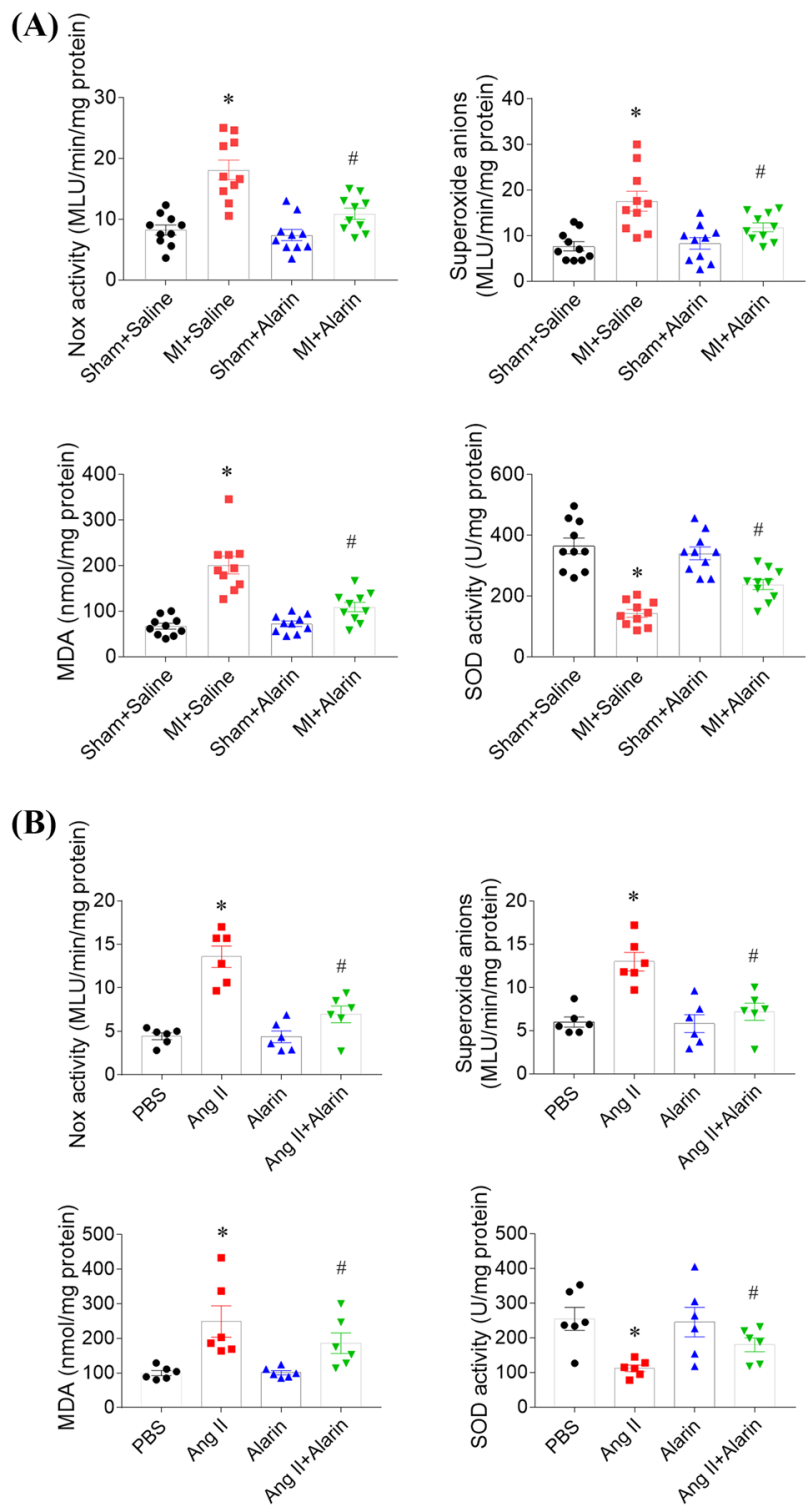
Alarin alleviated the oxidative stress in the heart of myocardial infarction (MI) rats and in the cardiac fibroblasts (CFs) induced by angiotensin (Ang) II. **A** Alarin reversed the increases of NADPH oxidase (Nox) activity, superoxide anions and malondialdehyde (MDA), and the decrease of superoxide dismutase (SOD) activity levels in the heart of MI rats. **B** Alarin reversed the increases of NADPH (Nox) activity, superoxide anions and malondialdehyde (MDA), and the decrease of superoxide dismutase (SOD) activity levels induced by Ang II in CFs. The results are expressed as mean ± SEM. ^*^p < 0.05 versus the Sham + Saline (**A**) or PBS (**B**) group; ^#^*p* < 0.05 versus the MI + Saline (**A**) or Ang II (**B**) group

MDA level was increased in in the heart of MI rats (201.24 ± 19.30 vs. 67.09 ± 6.53 mmol/mg protein), which was attenuated by alarin treatment (109.17 ± 10.42 vs. 201.24 ± 19.30 mmol/mg protein). SOD activity level was reduced in the heart of MI rats (142.81 ± 12.75 vs. 365.25 ± 25.88 U/mg protein), and alarin administration enhanced the decrease of SOD activity level in the heart of MI rats (237.82 ± 16.52 vs. 142.81 ± 12.75 U/mg protein) (Fig. 4A).

Nox activity level was increased in Ang II-treated CFs (13.57 ± 1.23 vs. 4.43 ± 0.39 MLU/min/mg protein). Alarin attenuated the increase of Nox activity level induced by Ang II in CFs (6.95 ± 0.95 vs. 13.57 ± 1.23 MLU/min/mg protein). The increase in the level of superoxide anion induced by Ang II in CFs (12.98 ± 1.07 vs. 5.98 ± 0.60 MLU/min/mg protein) was inhibited after alarin treatment (7.18 ± 0.98 vs. 12.98 ± 1.07 MLU/min/mg protein). MDA level was increased in Ang II-treated CFs (248.67 ± 45.21 vs. 99.67 ± 7.58 mmol/mg protein), which was attenuated by alarin treatment (186.17 ± 29.71 vs. 248.67 ± 45.21 mmol/mg protein). Ang II inhibited SOD activity level in CFs (112.17 ± 9.66 vs. 254.83 ± 33.13 U/mg protein), and alarin administration enhanced the decrease of SOD activity level in Ang II-treated CFs (180.17 ± 19.76 vs. 112.17 ± 9.66 U/mg protein) (Fig. 4B).

### Nox1 overexpression reversed the effects of alarin on inhibiting Ang II-induced CFs fibrosis

**Fig. 5:**
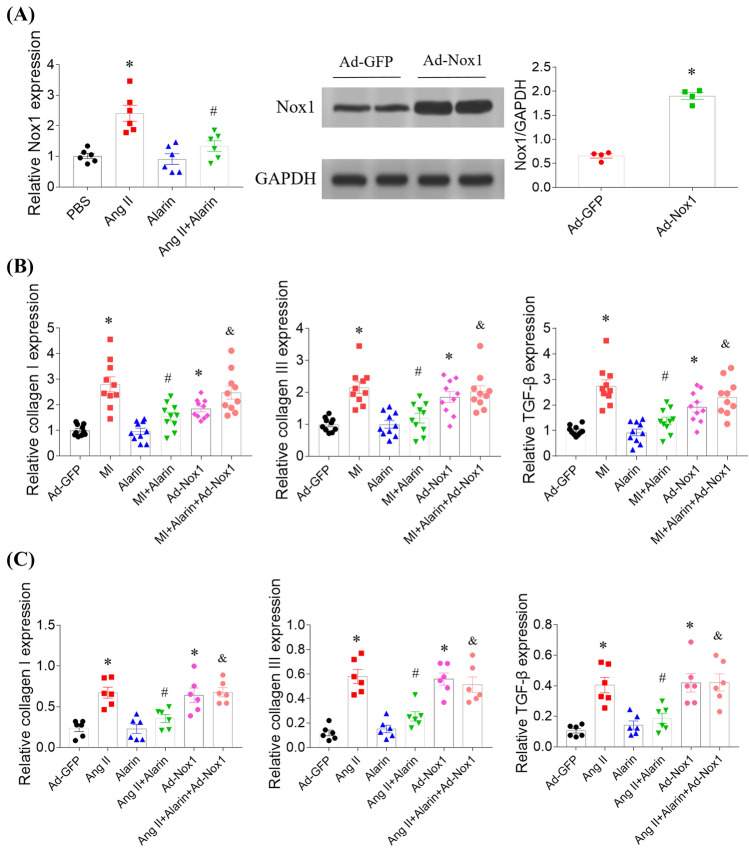
NADPH oxidase 1 (Nox1) overexpression reversed the effects of alarin on inhibiting cardiac fibrosis. **A** Alarin inhibited the increase of Nox1 in cardiac fibroblasts (CFs) induced by angiotensin (Ang) II. **B** Nox1 expression was significantly increased by overexpression. **C** Nox1 overexpression reversed the effects of alarin on inhibiting the increases of collagen I, collagen III and transforming growth factor-β (TGF-β) mRNA levels in the heart of myocardial infarction (MI) rats. **D** Nox1 overexpression reversed the effects of alarin on inhibiting Ang II-induced increases of collagen I, collagen III and TGF-β mRNA levels in CFs. The results are expressed as mean ± SEM. ^*^p < 0.05 versus the PBS (**A**) or Ad-GFP (**B**, **C** and **D**) group; ^#^*p* < 0.05 versus the Ang II (**A** and **C**) or MI (**B**) group; ^&^*p* < 0.05 versus the MI + Alarin (**B**) or Ang II + Alarin (**C**) group

The expression was increased in the CFs treated with Ang II, and this increase was inhibited by alarin administration (Fig. 5A). Nox1 expression in Ad-Nox1 treated CFs was 2.88 times of Ad-GFP treated CFs (Fig. 5B). Nox1 overexpression reversed the effects of alarin on attenuated the increases of collagen I, collagen III and TGF-β levels the herat of MI rats; while, Nox1 overexpression elevated the expression levels of collagen I, collagen III and TGF-β in the heart of rats (Fig. 5C). Nox1 overexpression reversed the effects of alarin on inhibiting Ang II-induced increases of collagen I, collagen III and TGF-β levels in CFs; while, Nox1 overexpression elevated the expression levels of collagen I, collagen III and TGF-β in CFs (Fig. 5D).

## Discussion

Alarin was identified as the fourth member of the galanin peptide family. It found that galanin level was correlated with humoral biomarkers with HF, pro-BNP and copeptin (Ozkaramanli Gur et al. 2017). Treatment with galanin (2–11) enhanced cell viability in a dose-dependent manner, attenuated cell apoptosis and excessive mitochondrial reactive oxygen species (ROS) production in response to oxidative stress in H9C2 cells (Timotin et al. 2017). We present found that alarin treatment improved HF and cardiac fibrosis in the rats, and inhibited Ang II-induced CFs fibrosis via attenuating oxidative stress.

Heart failure is a complex syndrome due to structural or functional cardiac disorders that impairs the ability of the ventricle to fill with or eject blood (Gonzalez et al. 2018; Patel and Deoghare 2015). It is characterized by progressive loss of contractility and ejection fraction, ventricular wall thinning, cardiac dilatation, dysregulated fluid homeostasis, cytokine and neurohumoral activation (Dorn and Molkentin 2004). There have been considerable advances in the pharmacological management of HF over the past 30 years. Despite effective medical interventions, mortality and morbidity remain substantial. In the present study, we found that EF, FS, LV + d*p*/d*t*_max_ and LVSP of LV were reduced in MI rats, which were reversed by alarin treatment. In MI rats, LVVS, LVVD, LVESD and LVEDD were elevated, and alarin administration inhibited these increases. These results indicated that alarin can attenuate MI-induced HF, and improved cardiac dysfunction of HF.

Heart failure is characterized by forming cardiac fibrosis. Myocardial interstitial fibrosis leaded to left ventricular dysfunction resulting in the development of HF (Gonzalez et al. 2018). Study showed that galanin receptor antagonist M40 effectively improved cardiac function of contraction, and attenuated cardiac fibrosis of MI-HF rats. However, it is not clear whether the relationship between alarin and fibrosis of heart in HF. In our present study, the results showed that the levels of collagen I, collagen III and TGF-β were increased in the heart of MI rats, and alarin treatment relieved these increases. Renin-angiotensin system plays an important role in the cardiac remodeling (Guimaraes et al. 2021). Ang II level was increased in MI-induced heart faliure model (Peng et al. 2020). Activated myofibroblasts express Ang II, and elevated Ang II consequently regulates the differentiation of myofibroblasts or the expression of extracellular matrix proteins (Cai et al. 2019). Hence, Ang II is commonly used in CFs to simulate cardiac fibrosis of MI (Wang et al. 2019a). We present found that the increases of collagen I, collagen III and TGF-β levels induced by Ang II treatment were attenuated after alarin administration in CFs. These results demonstrated that cardiac fibrosis were elevated in MI rats, and alarin improved fibrosis of the heart in HF rats.

So far, three types of galanin receptors have been described, including galanin receptor 1 (GalR1), GalR2 and GalR3. However, the receptor of alarin is still unknown (Sipkova et al. 2017). Previous study showed that Ala6-25 Cys is an antagonist of alarin (Wang et al. 2020), and C7 and M35 are the antagonists of galanin receptors (Zhang et al. 2019; Bhandari et al. 2010). We present found that Ala6-25 Cys, but not M35 or C7 reversed the attenuating effects of alarin on MI-induced cardiac fibrosis. In addition, Ala6-25 Cys reversed the effects of alarin on inhibiting the CFs fibrosis induced by Ang II. The results indicated that alarin produce the biological effects via an unknown receptor, but not a galanin receptor. Next, we found that administration of Ala6-25 Cys, M35 or C7 significantly reduced cardiac function of rats, indicating galanin family regulates the cardiac function on physiological state.

Reactive oxygen species (ROS), as important molecules in living organisms, are involved in many signaling pathways. As a key contributor to organ damage, oxidative stress is associated with various of diseases (Honda et al. 2019), including fibrosis of the heart (Kura et al. 2020). The increases of cell apoptosis and mitochondrial ROS production in H9C2 cells treated with hypoxia and reoxygenation were reduced exposure of short N-terminal galanin fragment 2–15 (Pisarenko et al. 2017). Antioxidants were showed to improve cardiac function and produce anti-fibrosis effect (Wang et al. 2019b; Liu et al. 2021). We present found that Nox activity, superoxide anion and MDA levels were increased, and SOD level was reduced in the heart of MI rats and CFs treated with Ang II, and alarin reversed these changes. So far, seven isoforms of Nox (Nox1–Nox5, dual oxidase 1 and dual oxidase 2) have been identified (Zhang et al. 2020). Our current study showed that Nox1 level was increased in Ang II-treated CFs, and this increase was inhibited by alarin administration. Moreover, Nox1 overexpression reversed the effects of alarin on inhibiting the increases of collagen I, collagen III and TGF-β in the heart of MI rats and CFs induced by Ang II. The expression levels of collagen I, collagen III and TGF-β were elevated by Nox1 overexpression in in the heart of rats and CFs. These results demonstrated that the oxidants and antioxidants were imbalanced in the heart of HF rats induced by MI, which could be reversed by alarin. Alarin could inhibit fibrosis of heart in HF via attenuating oxidative stress.

In addition to the mechanism of alarin acts directly on CFs, there are other pharmacological/physiological roles of alarin that are diminishing cardiac fibrosis independently of the effects on fibroblasts. Colocalization of galanin with vasoactive intestinal peptide, neuropeptide Y and substance P was observed in many nerve fibers of the heart (Zhu and Dey 1992). Alarin or its receptor may be expressed in the nerve fibers of the heart to regulate cardiac fibrosis via neuromodulation mechanism.

In conclusion, oxidative stress was enhanced in the heart of HF rats, which could be attenuated by alarin. Alarin functioned as an antioxidant to improve cardiac dysfunction, and attenuate cardiac fibrosis in HF rats and fibrosis in CFs treated with Ang II. Nox1 regulated the attenuating effects of alarin in HF and cardiac fibrosis.

## References

[CR1] Bhandari M, Kawamoto M, Thomas AC, Barreto SG, Schloithe AC, Carati CJ, Toouli J, Saccone GT (2010). Galanin receptor antagonist m35 but not m40 or c7 ameliorates cerulein-induced acute pancreatitis in mice. Pancreatology.

[CR2] Boughton CK, Patterson M, Bewick GA, Tadross JA, Gardiner JV, Beale KE, Chaudery F, Hunter G, Busbridge M, Leavy EM, Ghatei MA, Bloom SR, Murphy KG (2010). Alarin stimulates food intake and gonadotrophin release in male rats. Br J Pharmacol.

[CR3] Cai W, Zhong S, Zheng F, Zhang Y, Gao F, Xu H, Cai X, Lan J, Huang D, Shi G (2019). Angiotensin II confers resistance to apoptosis in cardiac myofibroblasts through the AT1/ERK1/2/RSK1 pathway. IUBMB Life.

[CR4] Dorn GW, Molkentin JD (2004). Manipulating cardiac contractility in heart failure: data from mice and men. Circulation.

[CR5] Fang P, Yu M, Shi M, Bo P, Zhang Z (2020). Galanin peptide family regulation of glucose metabolism. Front Neuroendocrinol.

[CR6] Fraley GS, Leathley E, Nickols A, Gerometta E, Coombs E, Colton S, Gallemore S, Lindberg A, Kofler B (2013). Alarin 6-25Cys antagonizes alarin-specific effects on food intake and luteinizing hormone secretion. Neuropeptides.

[CR7] Gan XB, Duan YC, Xiong XQ, Li P, Cui BP, Gao XY, Zhu GQ (2011). Inhibition of cardiac sympathetic afferent reflex and sympathetic activity by baroreceptor and vagal afferent inputs in chronic heart failure. PLoS ONE.

[CR8] Gan XT, Ettinger G, Huang CX, Burton JP, Haist JV, Rajapurohitam V, Sidaway JE, Martin G, Gloor GB, Swann JR, Reid G, Karmazyn M (2014). Probiotic administration attenuates myocardial hypertrophy and heart failure after myocardial infarction in the rat. Circ Heart Fail.

[CR9] Gonzalez A, Schelbert EB, Diez J, Butler J (2018). Myocardial interstitial fibrosis in heart failure: biological and translational perspectives. J Am Coll Cardiol.

[CR10] Guimaraes DA, Batista RIM, Tanus-Santos JE (2021). Nitrate and nitrite-based therapy to attenuate cardiovascular remodelling in arterial hypertension. Basic Clin Pharmacol Toxicol.

[CR11] Heineke J, Molkentin JD (2006). Regulation of cardiac hypertrophy by intracellular signalling pathways. Nat Rev Mol Cell Biol.

[CR12] Honda T, Hirakawa Y, Nangaku M (2019). The role of oxidative stress and hypoxia in renal disease. Kidney Res Clin Pract.

[CR13] Hu W, Fan X, Zhou B, Li L, Tian B, Fang X, Xu X, Liu H, Yang G, Liu Y (2019). Circulating alarin concentrations are high in patients with type 2 diabetes and increased by glucagon-like peptide-1 receptor agonist treatment: an consort-compliant study. Medicine (baltimore).

[CR14] Kong P, Christia P, Frangogiannis NG (2014). The pathogenesis of cardiac fibrosis. Cell Mol Life Sci.

[CR15] Kura B, Szeiffova Bacova B, Kalocayova B, Sykora M, Slezak J (2020). Oxidative stress-responsive microRNAs in heart injury. Int J Mol Sci.

[CR16] Liu C, Yang CX, Chen XR, Liu BX, Li Y, Wang XZ, Sun W, Li P, Kong XQ (2018). Alamandine attenuates hypertension and cardiac hypertrophy in hypertensive rats. Amino Acids.

[CR17] Liu Y, Li M, Du X, Huang Z, Quan N (2021). Sestrin 2, a potential star of antioxidant stress in cardiovascular diseases. Free Radic Biol Med.

[CR18] Madamanchi NR, Runge MS (2013). Redox signaling in cardiovascular health and disease. Free Radic Biol Med.

[CR19] Miko A, Balla P, Tenk J, Balasko M, Soos S, Szekely M, Brunner S, Kofler B, Petervari E (2014). Thermoregulatory effect of alarin, a new member of the galanin peptide family. Temperature (austin).

[CR20] Ohtsu H, Frank GD, Utsunomiya H, Eguchi S (2005). Redox-dependent protein kinase regulation by angiotensin II: mechanistic insights and its pathophysiology. Antioxid Redox Signal.

[CR21] Ozkaramanli Gur D, Sagbas M, Akyuz A, Guzel S, Alpsoy S, Guler N (2017). Role of sympathetic cotransmitter galanin on autonomic balance in heart failure: an active player or a bystander?. Anatol J Cardiol.

[CR22] Patel C, Deoghare S (2015). Heart failure: novel therapeutic approaches. J Postgrad Med.

[CR23] Peng Y, Li Y, Chen M, Song J, Jiang Z, Shi S (2020). High-dose nitrate therapy recovers the expression of subtypes alpha1 and beta-adrenoceptors and Ang II receptors of the renal cortex in rats with myocardial infarction-induced heart failures. BMC Cardiovasc Disord.

[CR24] Philip JL, Xu X, Han M, Akhter SA, Razzaque MA (2019). Regulation of cardiac fibroblast-mediated maladaptive ventricular remodeling by beta-arrestins. PLoS ONE.

[CR25] Pisarenko O, Timotin A, Sidorova M, Studneva I, Shulzhenko V, Palkeeva M, Serebryakova L, Molokoedov A, Veselova O, Cinato M, Boal F, Tronchere H, Kunduzova O (2017). Cardioprotective properties of N-terminal galanin fragment (2–15) in experimental ischemia/reperfusion injury. Oncotarget.

[CR26] Sag CM, Santos CX, Shah AM (2014). Redox regulation of cardiac hypertrophy. J Mol Cell Cardiol.

[CR27] Santic R, Fenninger K, Graf K, Schneider R, Hauser-Kronberger C, Schilling FH, Kogner P, Ratschek M, Jones N, Sperl W, Kofler B (2006). Gangliocytes in neuroblastic tumors express alarin, a novel peptide derived by differential splicing of the galanin-like peptide gene. J Mol Neurosci.

[CR28] Santic R, Schmidhuber SM, Lang R, Rauch I, Voglas E, Eberhard N, Bauer JW, Brain SD, Kofler B (2007). Alarin is a vasoactive peptide. Proc Natl Acad Sci USA.

[CR29] Sipkova J, Kramarikova I, Hynie S, Klenerova V (2017). The galanin and galanin receptor subtypes, its regulatory role in the biological and pathological functions. Physiol Res.

[CR30] Studneva I, Palkeeva M, Veselova O, Molokoedov A, Ovchinnikov M, Sidorova M, Pisarenko O (2019). Protective effects of a novel agonist of galanin receptors against doxorubicin-induced cardiotoxicity in rats. Cardiovasc Toxicol.

[CR31] Studneva I, Serebryakova L, Veselova O, Pal'keeva M, Molokoedov A, Ovchinnikov M, Konovalova G, Lankin V, Sidorova M, Pisarenko O (2019). Galanin receptors activation modulates myocardial metabolic and antioxidant responses to ischaemia/reperfusion stress. Clin Exp Pharmacol Physiol.

[CR32] Tallquist MD, Molkentin JD (2017). Redefining the identity of cardiac fibroblasts. Nat Rev Cardiol.

[CR33] Tarone G, Balligand JL, Bauersachs J, Clerk A, De Windt L, Heymans S, Hilfiker-Kleiner D, Hirsch E, Iaccarino G, Knoll R, Leite-Moreira AF, Lourenco AP, Mayr M, Thum T, Tocchetti CG (2014). Targeting myocardial remodelling to develop novel therapies for heart failure: a position paper from the Working Group on Myocardial Function of the European Society of Cardiology. Eur J Heart Fail.

[CR34] Timotin A, Pisarenko O, Sidorova M, Studneva I, Shulzhenko V, Palkeeva M, Serebryakova L, Molokoedov A, Veselova O, Cinato M, Tronchere H, Boal F, Kunduzova O (2017). Myocardial protection from ischemia/reperfusion injury by exogenous galanin fragment. Oncotarget.

[CR35] Wang L, Liu C, Chen X, Li P (2019). Alamandine attenuates longterm hypertensioninduced cardiac fibrosis independent of blood pressure. Mol Med Rep.

[CR36] Wang XB, Cui H, Du JB (2019). Potential therapeutic effect of SO(2) on fibrosis. Histol Histopathol.

[CR37] Wang Q, Deng F, Zhu D (2020). Superoxide anions modulate the effects of alarin in the paraventricular nucleus on sympathetic activity and blood pressure in spontaneously hypertensive rats. Neuropeptides.

[CR38] Yang C, Wu X, Shen Y, Liu C, Kong X, Li P (2020). Alamandine attenuates angiotensin II-induced vascular fibrosis via inhibiting p38 MAPK pathway. Eur J Pharmacol.

[CR39] Yue Y, Meng K, Pu Y, Zhang X (2017). Transforming growth factor beta (TGF-beta) mediates cardiac fibrosis and induces diabetic cardiomyopathy. Diabetes Res Clin Pract.

[CR40] Zhang Y, Gao Y, Li CY, Dong W, Dong Y, Li MN, Liu YN, Xu SL (2019). Galanin receptor 1 plays an antinociceptive effect via inhibiting PKA activation in the nucleus accumbens of rats with neuropathic pain. Physiol Res.

[CR41] Zhang Y, Murugesan P, Huang K, Cai H (2020). NADPH oxidases and oxidase crosstalk in cardiovascular diseases: novel therapeutic targets. Nat Rev Cardiol.

[CR42] Zhu W, Dey RD (1992). Distribution of the neuropeptide galanin in the cat heart and coexistence with vasoactive intestinal peptide, substance P and neuropeptide Y. J Mol Cell Cardiol.

